# Dataset on *Insilico* approaches for 3,4-dihydropyrimidin-2(1H)-one urea derivatives as efficient Staphylococcus aureus inhibitor

**DOI:** 10.1016/j.dib.2020.106195

**Published:** 2020-08-19

**Authors:** Abel Kolawole Oyebamiji, Ibrahim O. Abdulsalami, Banjo Semire

**Affiliations:** a Department of Basic Sciences, Adeleke University, P.M.B. 250, Ede, Osun State, Nigeria; b Department of Chemical Sciences, Fountain University, Osogbo, Nigeria; c Computational Chemistry Research Laboratory, Department of Pure and Applied Chemistry, Ladoke Akintola University of Technology, P.M.B. 4000, Ogbomoso, Oyo State, Nigeria

**Keywords:** 3,4-dihydropyrimidin-2(1H)-one Urea, Staphylococcus aureus, DFT, QSAR, Docking, inhibitor

## Abstract

Series of anti- Staphylococcus aureus were studied via quantum chemical method and several molecular descriptors were obtained which were further used to develop QSAR model using back propagation neural network method using MATLAB. More so, the molecular interaction observed between 3,4-dihydropyrimidin-2(1H)-one Urea Derivatives and *Staphylococcus aureus* Sortase (PDB ID Code: **2kid**) via docking was used as a screening tool for the studied compounds. The observed molecular compounds used in this work was also correlated to Lipinski rule of five and the developed QSAR model using selected descriptors from the optimized compounds was also examined for its predictability. Also, the observed molecular docking revealed the interaction between the studied complex.

**Specification Table**

**Table:** 

**Subject**	Computational Chemistry
**Specific subject area**	Drug Design
**Type of data**	Developed QSAR Model Equation Figure Table
**How data were** **acquired**	Spartan 14, Pymol 1.7.4.4, MATLAB, Autodock tool 1.5.6, AutoVina 1.1.2, Discovery Studio 2017
**Data format**	Analysed data (Developed, Observed and Calculated)
**Parameters for data collection**	B3LYP, 6–31G**, Gretl, Pymol 1.7.4.4, Discovery studio 2017R, Autodock tool 1.5.6 and Autodock vina 1.1.2.
**Description of data collection**	
**Data source location**	Computational Chemistry Research Laboratory, Department of Pure and Applied Chemistry, Ladoke Akintola University of Technology, P.M.B. 4000, Ogbomoso, Oyo State, Nigeria
**Data accessibility**	The observed and calculated data can be accessed with the data article

## 1. Value of the data

• Datasets obtained in this research will help the scientists to know the molecular descriptors which describe the anti- Staphylococcus aureus properties of 3,4-dihydropyrimidin-2(1H)-one Urea Derivatives.
• Data in this research will reveal the contribution of each calculated descriptor in the developed QSAR model.
• It also helps in predicting library of efficient drug-like compounds via the developed QSAR model.
• The ability of each observed compounds to inhibit Staphylococcus aureus via docking can also be understood.

## 2 Data description

The molecular compounds used in this work were displayed in Table 1. In this work, sixteen molecular compounds were subjected to density functional theory via B3LYP with the standard 6–31G** basis set for optimisation and the obtained molecular descriptors were reported for further investigation. 3,4-dihydropyrimidin-2(1H)-one Urea derivatives was extracted from the work done by Mukesh, 2015 [1].

**Table 1:** The Schematic diagram of 3,4-dihydropyrimidin-2(1H)-one urea derivatives [1].


**S. No**	**R**	
**A1**	2-F	ethyl 4-(4-(3-(2-fluorophenyl)ureido)phenyl)−1,4,5,6-tetrahydro-2-methyl-6-thioxopyridine-3-carboxylate
**A2**	2-Cl	ethyl 4-(4-(3-(2-chlorophenyl)ureido)phenyl)−1,4,5,6-tetrahydro-2-methyl-6-thioxopyridine-3-carboxylate
**A3**	2-CF_3_	ethyl 4-(4-(3-(2-(trifluoromethyl)phenyl)ureido)phenyl)−1,4,5,6-tetrahydro-2-methyl-6-thioxopyridine-3-carboxylate
**A4**	2-OCF_3_	ethyl 1,4,5,6-tetrahydro-2-methyl-6-thioxo-4-(4-(3-(2-(trifluoromethoxy)phenyl)ureido)phenyl)pyridine-3-carboxylate
**A5**	2-F, 6-CH3	ethyl 4-(4-(3-(2-fluoro-6-methylphenyl)ureido)phenyl)−1,4,5,6-tetrahydro-2-methyl-6-thioxopyridine-3-carboxylate
**A6**	2-F, 6-CF_3_	ethyl 4-(4-(3-(2-fluoro-6-(trifluoromethyl)phenyl)ureido)phenyl)−1,4,5,6-tetrahydro-2-methyl-6-thioxopyridine-3-carboxylate
**A7**	2-Cl, 6-CH3	ethyl 4-(4-(3-(2‑chloro-6-methylphenyl)ureido)phenyl)−1,4,5,6-tetrahydro-2-methyl-6-thioxopyridine-3-carboxylate
**A8**	2-Cl, 6-F	ethyl 4-(4-(3-(2‑chloro-6-fluorophenyl)ureido)phenyl)−1,4,5,6-tetrahydro-2-methyl-6-thioxopyridine-3-carboxylate
**A9**	3-CF_3_	ethyl 4-(4-(3-(3-(trifluoromethyl)phenyl)ureido)phenyl)−1,4,5,6-tetrahydro-2-methyl-6-thioxopyridine-3-carboxylate
**A10**	3-Cl, 4-F	ethyl 4-(4-(3-(3‑chloro-4-fluorophenyl)ureido)phenyl)−1,4,5,6-tetrahydro-2-methyl-6-thioxopyridine-3-carboxylate
**A11**	3,5-F	ethyl 4-(4-(3-(3,5-difluorophenyl)ureido)phenyl)−1,4,5,6-tetrahydro-2-methyl-6-thioxopyridine-3-carboxylate
**A12**	3,4-CH_3_	ethyl 1,4,5,6-tetrahydro-2-methyl-4-(4-(3-(3,4-dimethylphenyl)ureido)phenyl)−6-thioxopyridine-3-carboxylate
**A13**	4-F, 3-CH3	ethyl 4-(4-(3-(4-fluoro-3-methylphenyl)ureido)phenyl)−1,4,5,6-tetrahydro-2-methyl-6-thioxopyridine-3-carboxylate
**A14**	4-isopropyl	ethyl 1,4,5,6-tetrahydro-2-methyl-4-(4-(3-(4-propylphenyl)ureido)phenyl)−6-thioxopyridine-3-carboxylate
**A15**	4-CF_3_	ethyl 4-(4-(3-(4-(trifluoromethyl)phenyl)ureido)phenyl)−1,4,5,6-tetrahydro-2-methyl-6-thioxopyridine-3-carboxylate
**A16**	4–OCH_3_	ethyl 1,4,5,6-tetrahydro-4-(4-(3-(4-methoxyphenyl)ureido)phenyl)−2-methyl-6-thioxopyridine-3-carboxylate

Table 2 reveal the calculated molecular descriptors via density functional theory [2]. Series of calculated molecular parameter obtained were highest occupied molecular orbital (E_HOMO_), lowest unoccupied molecular orbital energy (E_LUMO_), band gap, molecular weight, Log P, Area, Ovality, polar surface area, polarisability, hydrogen bond donor (HBD), hydrogen bond acceptor (HBA) and number of rotatable bonds. Further investigation was conducted using Lipinski rule of five so as to determine the drug-likeness of the studied drug-like compounds [3].

**Table 2:** Calculated molecular descriptors from 3,4-dihydropyrimidin-2(1H)-one urea derivatives.

	E_HOMO_(eV)	E_LUMO_(eV)	BG(eV)	MW(amu)	LogP	AREA(A^2^)	VOL (A^3^)	OVALITY	PSA(A^2^)	Pol	HBD	HBA	PIC
A1*	−5.76	−1.35	4.41	428.49	2.32	438.04	411.06	1.64	71.73	73.68	4	7	−1
A2	−5.87	−1.4	4.47	444.94	2.72	442.77	418.99	1.64	67.75	74.31	4	7	−1
A3	−5.86	−1.42	4.44	478.5	3.08	460.51	437.19	1.65	66.84	75.79	4	7	−1.39
A4*	−5.78	−1.39	4.39	494.49	3.12	475.18	447.02	1.68	75.79	76.6	4	8	−1.47
A5	−5.78	−1.4	4.38	442.52	2.8	452.32	498.41	1.65	69.13	75.09	4	7	−1.77
A6	−5.85	−1.41	4.44	496.49	3.24	467.22	428.41	1.66	69.88	76.2	4	7	−1.74
A7	−5.79	−1.39	4.4	458.97	3.2	463.74	442.23	1.66	69.38	75.84	4	7	−1.60
A8	−5.86	−1.4	4.46	462.93	2.87	452.19	437.74	1.66	71.2	74.76	4	7	−1.81
A9	−5.83	−1.42	4.41	478.5	3.08	465.2	451.46	1.67	69.02	75.84	4	7	−1.60
A10	−5.81	−1.43	4.38	462.93	2.87	450.04	450.89	1.65	69.04	74.73	4	7	−1.77
A11	−5.84	−1.4	4.44	446.48	2.47	439.84	452.18	1.64	68.97	73.96	4	7	−1.95
A12*	−5.57	−1.42	4.15	438.55	3.13	467.3	424.58	1.67	69.12	76.24	4	7	−1.95
A13*	−5.61	−1.42	4.19	442.52	2.8	454.02	437.74	1.65	69.13	75.15	4	7	−1.81
A14	−5.59	−1.4	4.19	452.58	3.48	489.49	423.94	1.7	69.12	77.76	4	7	−1.92
A15	−5.85	−1.4	4.45	478.5	3.09	464.25	414.63	1.67	68.92	75.82	4	7	−1.30
A16*	−5.35	−1.41	3.94	440.52	2.03	459.04	441.91	1.66	76.05	75.55	4	8	−1.17

*Note: BG: Band gap; Vol: Volume; MW: molecular weight;* Log*P: Lipophilicity; PSA: polar surface area, Pol: Polarizability; HBD: Hydrogen bond Donor; HBA: Hydrogen bond Acceptor; PIC: negative* log *of inhibition concentration (IC_50_)*.

Table 3 showed the developed QSAR model using the calculated molecular descriptors using back propagation neural network (BPNN) via MATLAB software [4,5]. The developed QSAR model involved molecular weight, volume, polarisability, E_HOMO_ and Log P. This set of descriptors were chosen because they best described anti- Staphylococcus aureus activities of compounds used in this work than other calculated descriptors. The calculated correlation coefficient (R^2^) for the developed QSAR model was 0.930. The developed QSAR model was validated by considering several parameters such as Adjusted R^2^, Cross validation (C.VR^2^), P-Value, F-Value. Also, the molecular compounds used were divided in to two (Test set and Training set). The compounds used as training set were compound A2, A3, A5, A6, A7, A8, A9, A10, A11, A14, A15 and the compounds used as test set were A1, A4, A12, A13 and A16.

**Table 3:** Developed QSAR model for 3,4-dihydropyrimidin-2(1H)-one Urea derivatives.

Equation	F	P-value	R^2^	Adj. R^2^	C.VR^2^	MSE
IC_50_ = −2209.75 - 0.0380508(MW) - 4.15718(Vol) + 51.7411(Pol) - 21.4175(E_HOMO_) + 1.03509 (LogP)	13.36	*P* < 0.0001	0.930	0.860	0.999	0.005

**Table 4:** Correlation between the observed IC_50_ and predicted IC_50_.

	PIC_50_	BPNN	Residue
A1*	5.0132	4.988758	0.024442
A2	5.0132	4.986026	0.027174
A3	4.6020	4.59819	0.00381
A4*	4.5228	4.495399	0.027401
A5	4.2218	4.202829	0.018971
A6	4.2596	4.256674	0.002926
A7	4.3979	4.389545	0.008355
A8	4.1870	4.170594	0.016406
A9	4.3979	4.369175	0.028725
A10	4.2218	4.192853	0.028947
A11	4.0457	4.040972	0.004728
A12*	4.0457	4.016582	0.029118
A13*	4.1870	4.158285	0.028715
A14	4.0705	4.055939	0.014561
A15	4.6989	4.674892	0.024008
A16*	4.8239	4.819643	0.004257

⁎ Test Set.

**Fig. 1:**
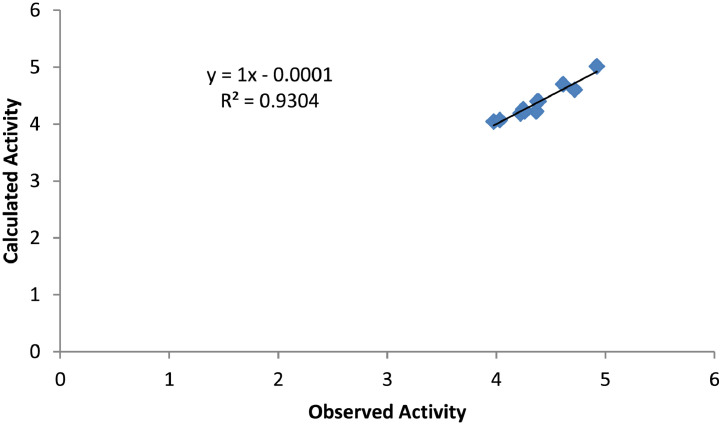
Graphical representation showing the correlation between calculated activity and observed activity.

Therefore, table 4 reveal the effectiveness of the developed model shown in Table 3. Also, correlation between the observed and the predicted inhibition concentration was displayed in Fig. 1. More so, five (5) molecular compounds were proposed and the IC_50_ were predicted using the developed QSAR model (Table 5).

**Table 5:** Structure for proposed compounds with the biological activities.


	R	IC_50_
**1**	CH_3_	1.23
**2**	CH_2_F	1.56
**3**	CHF_2_	2.06
**4**		22.89
**5**		11.29
**6**		12.09

Series of 3,4-dihydropyrimidin-2(1H)-one Urea Derivatives were docked against *Staphylococcus aureus* sortase and the binding affinity, inhibition constant as well as amino residues observed in the interaction between 3,4-dihydropyrimidin-2(1H)-one Urea Derivatives and *Staphylococcus aureus* sortase (PDB ID Code: **2kid**) [6] were displayed in Table 6. The residues involved in the interaction were displayed in SI.

**Table 6:** Interactions between 3,4-dihydropyrimidin-2(1H)-one Urea Derivatives and *Staphylococcus aureus* sortase (PDB ID Code: **2kid**).

Comp	Scoring (kcal/mol)	K (μM)	Amino Acid Residues
A1	−7.2	1.89748 × 10^5^	THR-121, TYR-187, ASP-185, ILE-123
A2	−6.9	1.14354 × 10^5^	VAL-168, TRP-194, VAL-166, ARG-197, HIS-120
A3	−7.6	3.72744 × 10^5^	TYR-187, ILE-123, ASP-185, TRP-194
A4	−7.3	2.24640 × 10^5^	THR-121, ILE-123, TYR-187, ASP-185
A5	−7.4	2.65947 × 10^5^	TYR-187, ASP-185, ILE-123
A6	−6.9	1.14354 × 10^5^	TYR-187, THR-121, ILE-123, TRP-194, PHE-122
A7	−6.3	4.1534 × 10^4^	ASP-186, ASP-185, ILE-123
A8	−7.2	1.89748 × 10^5^	TYR-187, ILE-123, ASP-185
A9	−7.4	2.65947 × 10^5^	TYR-187, ASP-185, ILE-123
A10	−6.8	9.6593 × 10^4^	TYR-187, TRP-194, ASP-185, ILE-1123
A11	−6.9	1.14354 × 10^5^	ARG-197, VAL-168, THR-164, ASP-165, TRP-194, HIS-120
A12	−7.5	3.14849 × 10^5^	TYR-187, ASP-185, ILE-123
A13	−7.2	1.89748 × 10^5^	TRP-194, TYR-187, ILE-123, ASP-185
A14	−7.4	2.65947 × 10^5^	ILE-123, TYR-187
A15	−7.4	2.65947 × 10^5^	PRO-91, ALA-92, THR-93, ILE-199, ILE-182, VAL-168, ARG-197
A16	−7.0	1.35382 × 10^5^	ASP-185, ILE-123, TYR-187
Cephalexin	−5.7	1.5085 × 10^4^	ILE-123; ASP-185; ASP-186; TYR-187

Proposed Compounds			

1	−5.8	1.7859 × 10^4^	GLN-64; LYS-71; VAL-72; GLY-147; LYS-62; ASN-148
2	−6.3	4.1534 × 10^4^	LYS-162; ASP-165; ALA-92; ALA-104; LEU-169; ILE-182; ALA-118
3	−6.5	5.8213 × 10^4^	ASP-165; THR-164; LYS-162; PRO-163; ALA-92; ALA-104; LEU-104; LEU-169; ILE-182; ALA-118
4	−6.5	5.8213 × 10^4^	TYR-187; THR-121; PHE-122
5	−5.6	1.2742 × 10^4^	ILE-65; PRO-89
6	−6.3	4.1534 × 10^4^	ASP-185; THR-121; TYR-187; TRP-194; PHE-122

More so, the interaction between the proposed compounds and *Staphylococcus aureus* sortase (PDB ID Code: **2kid**) were displayed in Table 6. The molecular interaction between the molecular compounds used and the receptor were displayed in SII.

## 3 Experimental design, materials, and methods

In this work, series of vital materials (Software) were used to accomplish this research [7]. Spartan’14 was used to optimised 3,4-dihydropyrimidin-2(1H)-one Urea derivatives studied in this work. The density functional theory used for the optimisation was achieved using three-parameter B3LYP that comprises Becke's gradient exchange correction [8,9], Lee, Yang, as well as Parr correlation functional [10]. It was through this that several molecular descriptors were obtained to develop QSAR model using BPNN via MATLAB software. Also, docking was accomplished using pymol 1.7.4.4 software. It was used for treating (removal of foreign compounds) downloaded *Staphylococcus aureus* Sortase (PDB ID Code: **2kid**) from protein data bank (www.rcsb.org). Also, the treated *Staphylococcus aureus* Sortase (PDB ID Code: **2kid**) was subjected to autodock tool 1.5.6 so as to locate the binding sites in the receptor and convert the receptor as well as the ligand to the format which will acceptable by autodock vina 1.1.2 that will do the docking calculation. The use of autodock tool 1.5.6 require the use of commands in order to accomplish the docking calculation; to execute the calculation, vina –config conf.txt –log.txt was used. Also, vinasplit –input out.pdbqt was used to split the calculated binding affinity according to the energy of each conformation. The observed grid box was as follows: centre (*X* = 0.677, *Y* = 0.25, *Z* = −1.245) and size (*X* = 64, *Y* = 52, *Z* = 56).

## Funding

This research received no external funding**.**

## Declaration of Competing Interest

The authors declare that they have no conflict of interest.

## Appendix Supplementary materials

Image, application 1

Image, application 2
