# Supplementary material for: Dataset on Insilico approaches for 3,4-dihydropyrimidin-2(1H)-one urea derivatives as efficient Staphylococcus aureus inhibitor
Source: Data Brief. 2020 Aug 19;32:106195. doi: 10.1016/j.dib.2020.106195 (PMC7476855; doi:10.1016/j.dib.2020.106195)
Supplement: Supplementary file 1 [file mmc1.docx]

| 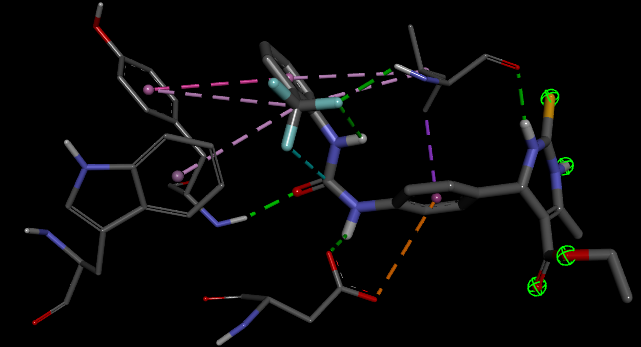 | 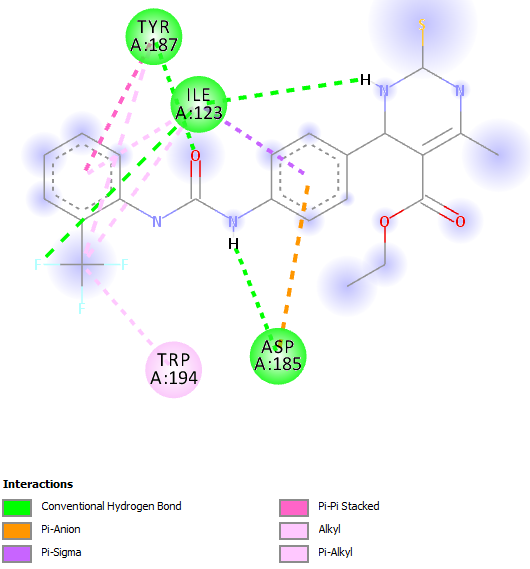 |
| --- | --- |
| 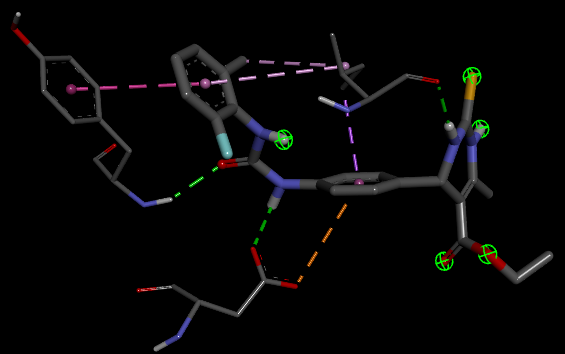 | 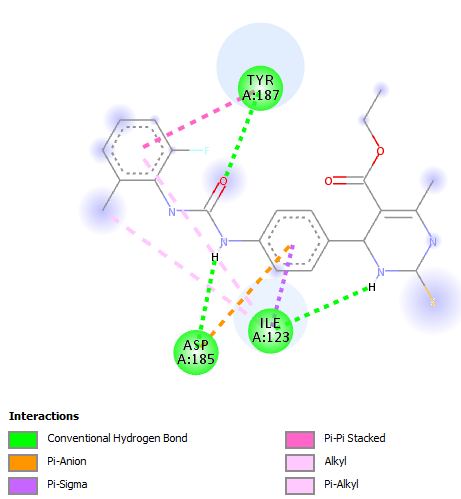 |
| 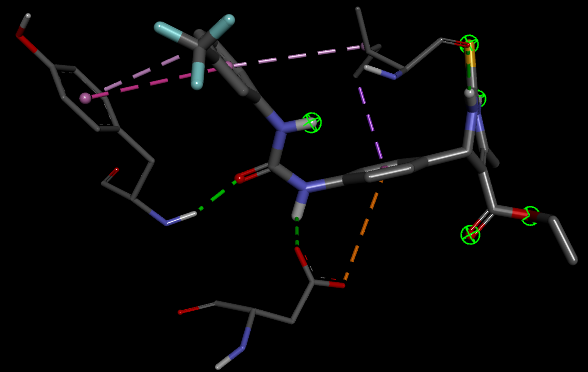 | 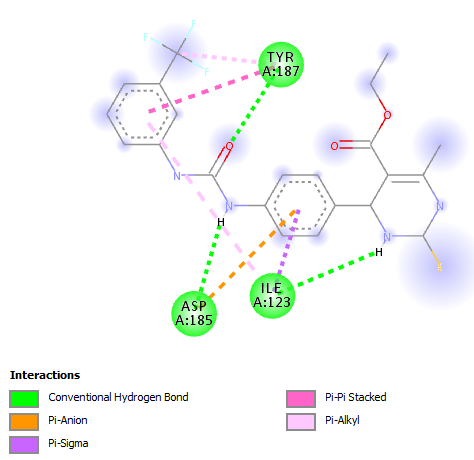 |
| 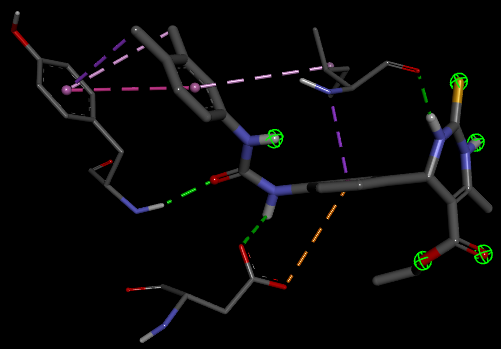 | 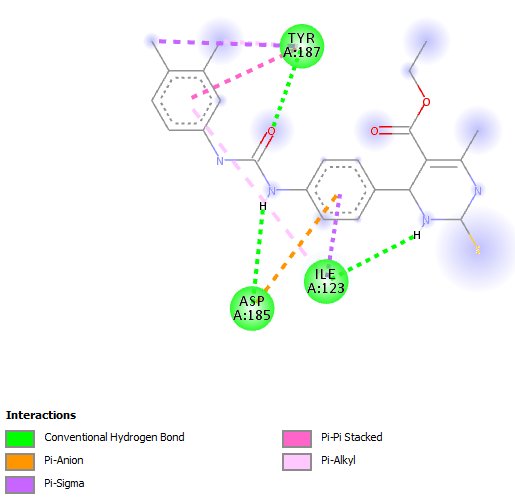 |
| 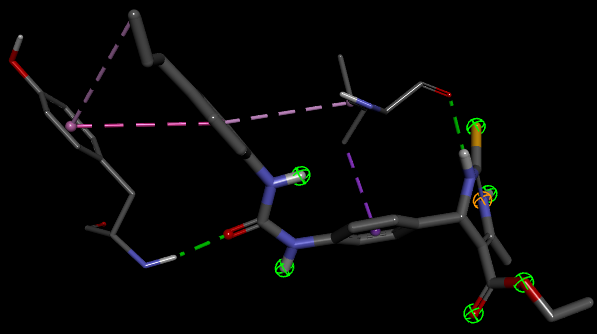 | 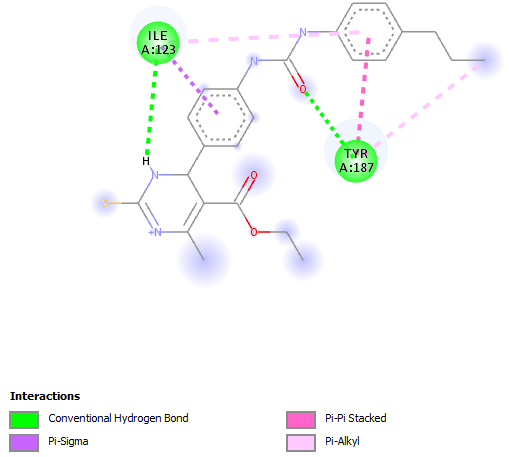 |
| 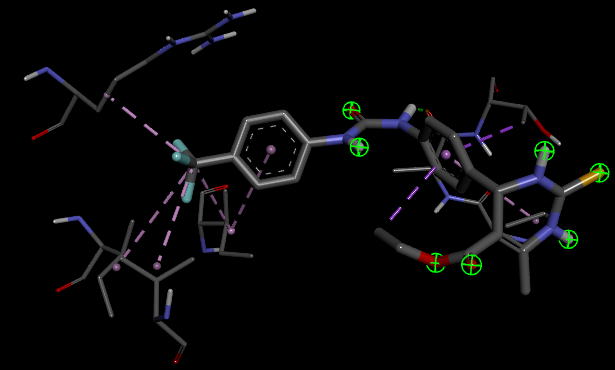 | 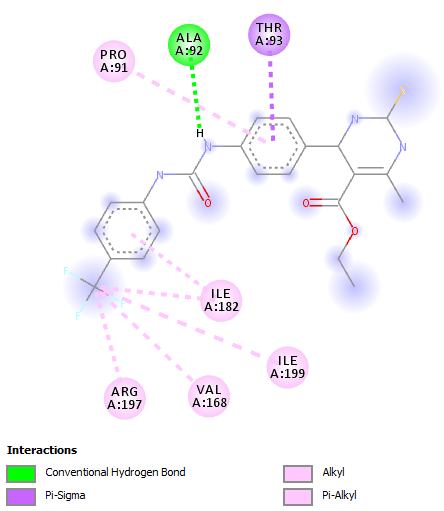 |

**Figure SI:** Molecular interaction of compound **A3, A5, A9, A12, A14** and **A15** with **2kid** respectively.

| 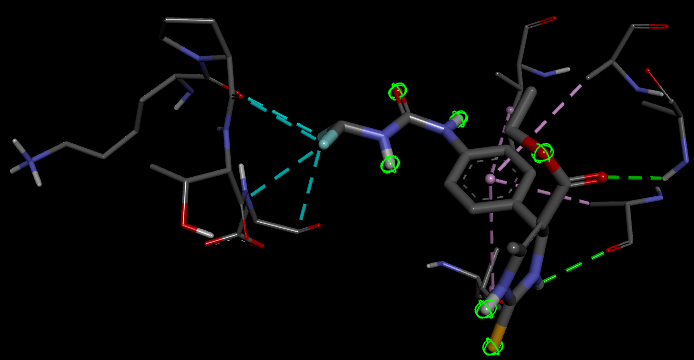 | 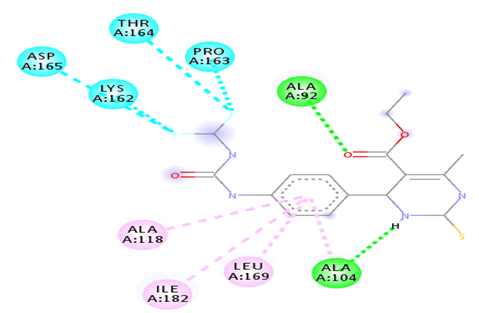 |
| --- | --- |
| 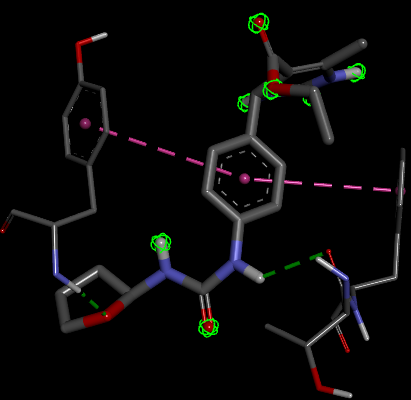 | 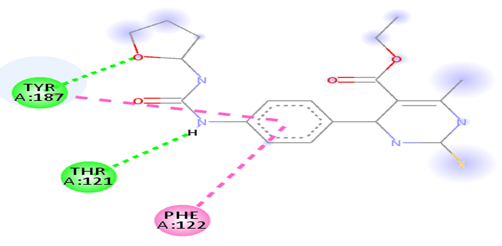 |

**Figure SII:** Molecular interaction of proposed compound **3** and **4** with **2kid**
